# The Curability of Consumption—Remarks on Prof. Jacoud’s New Book

**Published:** 1885-07

**Authors:** 


### THE CURABILITY OF CONSUMPTION.

An English translation of Dr. Jaccoud's work on "The Curability and Treatment of Pulmonary Phthisis" has been issued, and the *Journal of the A. M. A.* regards it as "one of the most important medical events of the year," devotes the whole of its editorial space (June 27) to a review of the book, and discusses the subject in a very learned way.

It seems that climatic influences are more relied upon than any purely medical treatment; and that the author does not claim to have found any specific course of treatment, nor does he make the preposterous claim that all cases are amenable to successful treatment. We gather from Dr. Davis' reviews that the book was written to show simply, that, under certain conditions, the affection is curable. It is very certain that American physicians study too little the influence of climate on health and disease, and, in that respect, are far behind their European brethren. In this connection it is interesting to note in the Proceedings of the American Climatological Association, a meeting of which was recently held in New York, that steps are being taken to make practical tests and experiments as to the real value of the climate—of certain localities in this country, which have, somehow, gotten the reputation of being "salubrious," to the end that physicians may act more intelligently in advising patients to go to this place or that. It is too much the custom of physicians to send patients away—any where—to get rid of them, if the cases are difficult, incurable or unprofitable. Certain it is that pulmonary phthisis is *the* opprobrium of medicine; and there is no subject on which there is a greater diversity of opinion, especially as regards its curability. That spontaneous recoveries have taken place in phthisical patients, even after the formation of cavities, cicatrices in the lung, found on *post mortem* examination, have often testified.

